# Statistics of thermomagnetic breakdown in Nb superconducting films

**DOI:** 10.1038/s41598-019-39337-5

**Published:** 2019-03-06

**Authors:** S. Blanco Alvarez, J. Brisbois, S. Melinte, R. B. G. Kramer, A. V. Silhanek

**Affiliations:** 1 Experimental Physics of Nanostructured Materials, Q-MAT, CESAM, Université de Liège, B-4000 Sart Tilman, Belgium; 2 Institute of Information and Communication Technologies, Electronics and Applied Mathematics (ICTM), Institut de la Matière Condensée et des Nanosciences (IMCN), Université catholique de Louvain, 1348 Louvain-la-Neuve, Belgium; 3 Institut Néel, CNRS, Université Grenoble Alpes, 38042 Grenoble, France

**Keywords:** Superconducting properties and materials, Superconducting properties and materials

## Abstract

Superconductors are well known for their ability to screen out magnetic fields. In type-II superconductors, as the magnetic field pressure is progressively increased, magnetic flux accumulates at the periphery of the sample, very much like charges accumulate in a capacitor when voltage is increased. As for capacitors, exceeding certain threshold field causes the blocked magnetic flux to abruptly penetrate into the sample. This phenomenon, triggered by a thermomagnetic instability, is somewhat analogous to the dielectric breakdown of the capacitor and leaves behind a similar Lichtenberg imprinting. Even though electrical breakdown threshold has been extensively studied in dielectrics, little information is known about the statistical distribution of the thermomagnetic breakdown in superconductors. In this work, we address this problem by performing magneto-optical imaging experiments on a Nb film where nanometric heating elements are used to rapidly erase the magnetic history of the sample. We demonstrate that the size and shape distributions of avalanches permits to unambiguously identify the transition between two regimes where either thermal diffusivity or magnetic diffusivity dominates. Clear criteria for discriminating athermal dynamic avalanches from thermally driven avalanches are introduced. This allows us to provide the first precise determination of the threshold field of the thermomagnetic breakdown and unveil the details of the transition from finger-like magnetic burst to dendritic branching morphology. These findings open a new avenue in the interdisciplinary exploration of catastrophic avalanches through non destructive repeatable experiments.

## Introduction

In the general theory of failure, if seemingly identical devices are subjected to a monotonically growing stress parameter *σ* under the same environmental conditions, they will not fail at exactly the same threshold stress *σ*_th_. The distribution of *σ*_th_ may arise from uncontrolled or imperceptible differences between the processed devices. In this case, the determination of the probability density function (PDF) associated to *σ*_th_ is essential for quantifying the reliability of the device and can be regarded as a manifestation of its reproducibility. Interestingly, even if all devices were *de facto* identical, the inherent stochasticity of the considered failure mechanism would still leave its imprint in a characteristic finite spread PDF of *σ*_th_. This analysis has far reaching pluridisciplinary implications touching a large diversity of phenomena, such as dielectric breakdown where *σ* corresponds to the bias voltage^1^, electromigration with *σ* being associated with the current density stimulating atom diffusion^2^, avalanches where *σ* represents the angle of the slope^3^, or even popcorn explosion with *σ* being the temperature of the hot plate^4^.

In the examples listed above, exceeding *σ*_th_ may have dramatic consequences leading to irreversible changes in the system, which requires large amount of replicas if we are interested in revealing the statistics (typically costly and time consuming). Interestingly, superconducting materials offer an ideal playground to investigate the PDF of the threshold stress *σ*_th_ in the very same sample, thus ruling out completely the spreading factors associated to unavoidable variations in the replicas of the system. Indeed, thin films superconductors of type II allow the penetration of quantum magnetic flux units from the sample’s borders, which builds up a flux gradient as a consequence of the uniform pinning landscape encountered along their path. Here, the analogy between quantum flux units and sand grains may be practical to imagine the inverted roof profile resulting from the magnetic field penetration. As for the sandpile, there is a critical field slope beyond which an avalanche can be triggered^5,6^. When these avalanches involve a small number of flux quanta, they help to relax the system and reestablish the critical slope. However, large avalanches produce local heat which in turn reduces the critical angle and promotes even further flux displacement. This positive gain feedback loop leads to sudden bursts of flux sometimes leaving a multibranch dendritic footprint of magnetic field into the sample, very much like the tracks left behind by a lightning strike during a dielectric breakdown^1,7^. In the superconducting system, the driving stress parameter is the magnetic field *H*, applied perpendicularly to the plane of the film, and the threshold stress is noted *H*_th_. An excellent review concerning the formation of magnetic flux avalanches and their statistical properties can be found in ref.^8^.

It might come as a surprise to learn that as of today, there are neither theoretical predictions nor experimental investigations of the statistical distribution of *H*_th_. On the one hand, while theoretical simulations have been successfully used to emulate flux avalanches and deduce some of their properties^9,10^, they still fail to capture the stochasticity of the process. On the other hand, the lack of experimental information can be tracked back to the techniques used to estimate *H*_th_. Magnetization measurements offer the possibility to identify *H*_th_ as the magnetic field at which the first magnetization jump is detected. Unfortunately, these studies based on Hall probe arrays and global magnetization measurements conceal information about different nucleation spots in the sample, the size of individual avalanches and their morphology. Alternatively, a more reliable and direct approach consists in visualizing the magnetic field profile through the magneto-optical (MO) imaging technique. In all cases, resetting the sample to the initial condition requires to warm it up above the superconducting transition temperature *T*_c_ and to cool it down in zero field back to the working temperature *T*_0_. Since flux avalanches only develop at *T*_0_/*T*_c_ < 0.5, the typical resetting takes several minutes, thus severely undermining the possibility to collect enough data to reliably determine the PDF.

In this work, we lift this technical limitation by introducing planar nanoheaters allowing to cycle the temperature within a short time frame, therefore facilitating the acquisition of the MO data necessary to estimate the PDF of magnetic flux avalanches. Strikingly, even though the MO imaging technique is insensitive to the local temperature and does not provide information on the time evolution of the events, the obtained PDF unravels a regime of small avalanches characteristic of a rapid evacuation of heat, separated from a regime of larger avalanches resulting from a substantial magnetic diffusivity and reduced heat spreading. This precious finding allows in turn for a precise determination of the threshold magnetic field *H*_th_ and its distribution probability with unprecedented resolution. Repeating the statistical analysis at several temperatures, we highlight the presence of two distinct *H*_th_ distributions corresponding to small and large avalanches, peaking respectively at low and high magnetic fields and coexisting at intermediate temperatures.

## Experimental details

**Figure 1:**
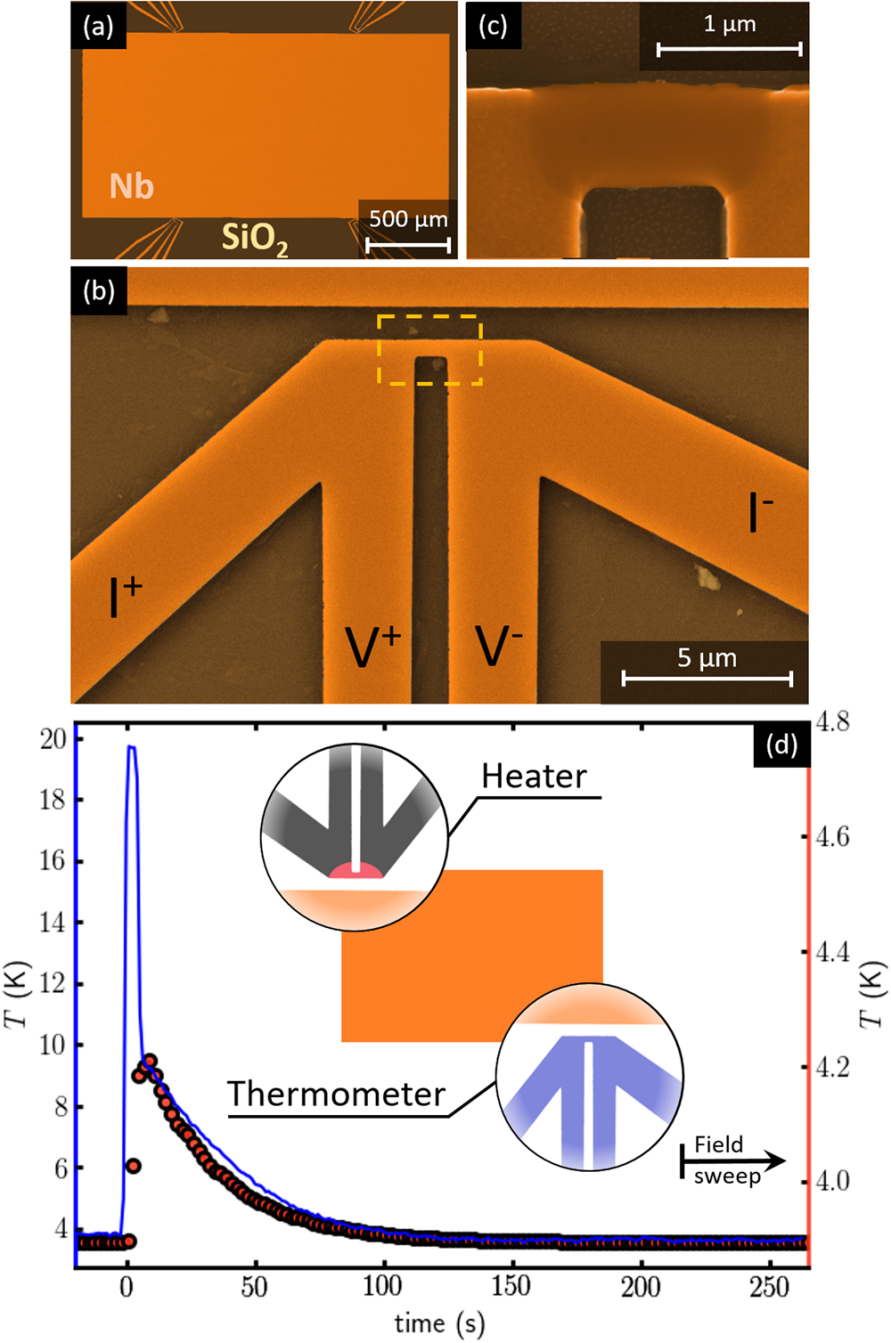
Sample layout and local thermometry configuration. Scanning electron microscopy details of the investigated sample. (**a**) Layout of the rectangular Nb film with four thermal elements symmetrically placed along the long sides. (**b**) Zoom-in on one of the as-fabricated Nb thermal elements, highlighting the central bridge. (**c**) Most of the voltage drop takes place in the short Nb bridge made non-superconducting via electroannealing, corresponding to the dashed yellow rectangle in panel (b). The inset in panel (d) shows the configuration used for tracking the temperature of the substrate with one thermal element used as nanothermometer, while exciting the diagonally opposed nanoheater with a 7 mA current during 5 s. The blue continuous line in panel (d) shows the time evolution of the sample temperature detected by the nanothermometer (left axis) and the red dots correspond to the temperature change detected in the cold finger thermometer (right axis). MO measurements start with a magnetic field sweep once the temperature of the substrate has been stabilized, as indicated by the black arrow.

The experiments were conducted on a 100 nm-thick Nb film prepared in a home-built electron beam UHV evaporator on top of a monocrystalline (100) Si substrate with a 100 nm-thick thermally grown SiO2 layer. Details concerning the evaporation parameters can be found in ref.^11^. The choice of material has been largely motivated by the extensive investigations already reported in the literature of magnetic flux avalanches in Nb^12–17^. The thin film was patterned using electron beam lithography followed by a reactive ion etching process. The layout of the sample investigated in this work is summarized in Fig. 1. It consists of a rectangular Nb film of 2 × 1 mm² and four thermal elements made of the same material symmetrically placed along the long side of the rectangular sample [Fig. 1(a)]. The small dimensions of the 4-wire thermal elements, shown in Fig. 1, are intended to minimize the perturbations on the magnetic field distribution and consequently, on the flux penetration into the rectangular Nb film (see Supplementary Information).

In their as-fabricated state, the thermal elements can only be employed as nanoheaters when fed with currents above the superconducting critical current of Nb. However, by performing a controlled electroannealing process^18^, we are able to locally change the material properties and render them non-superconducting. Figure 1(c) shows a scanning electron microscopy image, corresponding to the yellow dashed rectangle of panel (b), after the central part of the nanoheater has been modified by electroannealing. During this process the normal state resistance changes from 5 Ω in the as-fabricated state to 50 Ω after electroannealing. Although one single nanoheater should suffice, they are fragile against electrostatic discharges and chances of obtaining a successful operational device scale with their number. In addition, each nanoheater can also be used as a local thermometer allowing to monitor the temperature evolution of the sample during each cycle. In Fig. 1(d) (blue curve), we record the *T* modulations picked up in one nanothermometer caused by the heating produced by a 5 seconds current pulse in the diagonally opposed nanoheater (see inset). The sample temperature reaches a maximum value of 20 K, well above the superconducting critical temperature of Nb (*T*_c_ = 9.0 K). Once the excitation is switched off, the recovery to the initial temperature is characterized by two well distinguished relaxation times: a rapid exponential decay of the substrate temperature to the temperature of the cold finger (*τ*_1_ = 0.7 s) followed by a slower thermalization of the cold finger to the bath temperature (*τ*_2_ = 32 s). The slow relaxation of the latter is corroborated by the temporal evolution of the cold finger thermometer, as shown by the red dots in Fig. 1(d). It is worth noting that both decay times are much larger than the intrinsic relaxation time of the Si substrate once the heater is switched off, estimated as^19,20^ *τ* = *l*^2^*ρC*/*κ* ≈ 10^−6^ s, taking the density *ρ* = 2.33 g.cm^−3^, *l* = 1 mm, the heat capacity *C* = 1.6 × 10^−5^ J.K^−1^ and the thermal conductivity *κ* = 2.2 W.cm^−1^.K^−1^. In other words, the high thermal conductivity of the substrate *κ* combined with a low heat transfer coefficient *h* between the substrate and the cold finger, leads to a large thermal healing length $\delta =\sqrt{\kappa t/h}$, with *t* the substrate thickness. This ensures a rather uniform heating of the entire sample when using a single nanoheater.

Direct visualization of the magnetic flux landscape was obtained by MO imaging. This technique is based on the Faraday rotation of linearly polarized light in a 3 *μ*m-thick Bi-doped yttrium iron garnet (indicator) with in-plane magnetic domains, placed on top of the investigated sample^21^. Since the rotation of polarization is proportional to the local magnetic field *B*_*z*_ at the indicator, the use of an analyzer oriented perpendicularly to the initial direction of polarization results in images where the intensity is proportional to *B*_*z*_. The images are acquired with a CCD camera and have a pixel size of 1.468 × 1.468 *μ*m^2^. Post-image processing was done to remove the inhomogeneous illumination and field-independent background, using the ImageJ software. More information about the MO imaging setup can be found in ref.^22^. Low temperature MO measurements are performed in a closed-cycle cryostat and the external magnetic field was applied through a copper coil with resistance *R* = 22.1 Ω and inductance *L* = 26 mH. MO imaging allows for the detection of small magnetic field changes^22^ (~10 *μ*T). This value is to be compared with the smallest magnetization change 50 *μ*T picked up by a superconducting quantum interference device (SQUID) with typical sensitivity of 10^−8^ emu, on the entire sample^23^. More importantly, unlike such specimen-average measurements, MO imaging allows to record spatial maps of the magnetic flux and thus obtain direct information on the location and size of each event.

## Quantitative criterion for thermomagnetic breakdown

Let us begin by recalling that there are two well distinguished regimes of magnetic flux penetration in hard type-II superconductors: a smooth flux penetration described by the critical state model^15,24^ and a regime dominated by thermomagnetic instabilities^25^. In the former case, the thermal diffusion coefficient *D*_t_ = *κ*_Nb_/*C*_Nb_, where *κ*_Nb_ is the thermal conductivity of the superconducting material and *C*_Nb_ its specific heat, exceeds the magnetic diffusion coefficient *D*_m_ = *c*^2^/4*πσ*, with *σ* the normal state electrical conductivity and *c* the speed of light. In other words, the heat generated by the magnetic flux motion is rapidly distributed in the entire sample before further flux motion develops. In the opposite situation where ${D}_{{\rm{m}}}\gg {D}_{{\rm{t}}}$, rapid magnetic flux diffusion takes place while leaving behind a trail of overheated material which slowly diffuses.

The two main distinctive features of thermally driven avalanches are (i) a supersonic flux propagation, and (ii) a high temperature wake left along their paths. Most of magneto-optical studies reporting on avalanches produced by thermomagnetic instabilities are based on static pictures and hence they lack the time resolution needed to track the evolution of the flux propagation, neither are they sensitive to the local temperature distribution. Therefore, conclusions as to whether an avalanche belongs to one regime or the other are based on seemingly speculative arguments such as their size. This approximate conjecture is nevertheless valid for extreme cases of large flux burst, but certainly becomes unreliable for small avalanche sizes. The associated uncertainty is particularly prominent for the case of the first avalanche defining the transition point ${D}_{{\rm{m}}} \sim {D}_{{\rm{t}}}$ at a threshold magnetic field *H*_th_ and therefore, early determinations of *H*_th_ are inaccurate.

We will now show that a rigorous statistical analysis of the avalanche distribution permits to overcome this deficiency of the MO imaging technique and to precisely distinguish a dynamically driven (nearly isothermal) avalanche from thermally driven (adiabatic) flux penetration. The measurement protocol consists in a zero-field cooling to the working temperature *T*_0_, followed by a step-wise *δH* = 0.25 Oe magnetic field sweep between *H*_min_ < *H*_th_ and *H*_max_ > *H*_th_. Subsequently, the magnetic field is turned off and a delay much larger than the time constant of the coil (~1 ms) is imposed before heating the whole substrate with one nanoheater. The nanoheater is active during 5 s, ensuring a complete removal of the magnetic history of the sample, as directly verified by MO images inspection. Once the heater is switched off, a delay of 210 seconds is respected in order to guarantee that the system has recovered the initial temperature *T*_0_. This time has been experimentally determined by warming up with one heating elements and monitoring the temperature change in the diagonally opposed heating element, as shown in Fig. 1(d). This procedure has been repeated 2000 times thus collecting about 1.5 × 10^5^ images for a given temperature.

**Figure 2:**
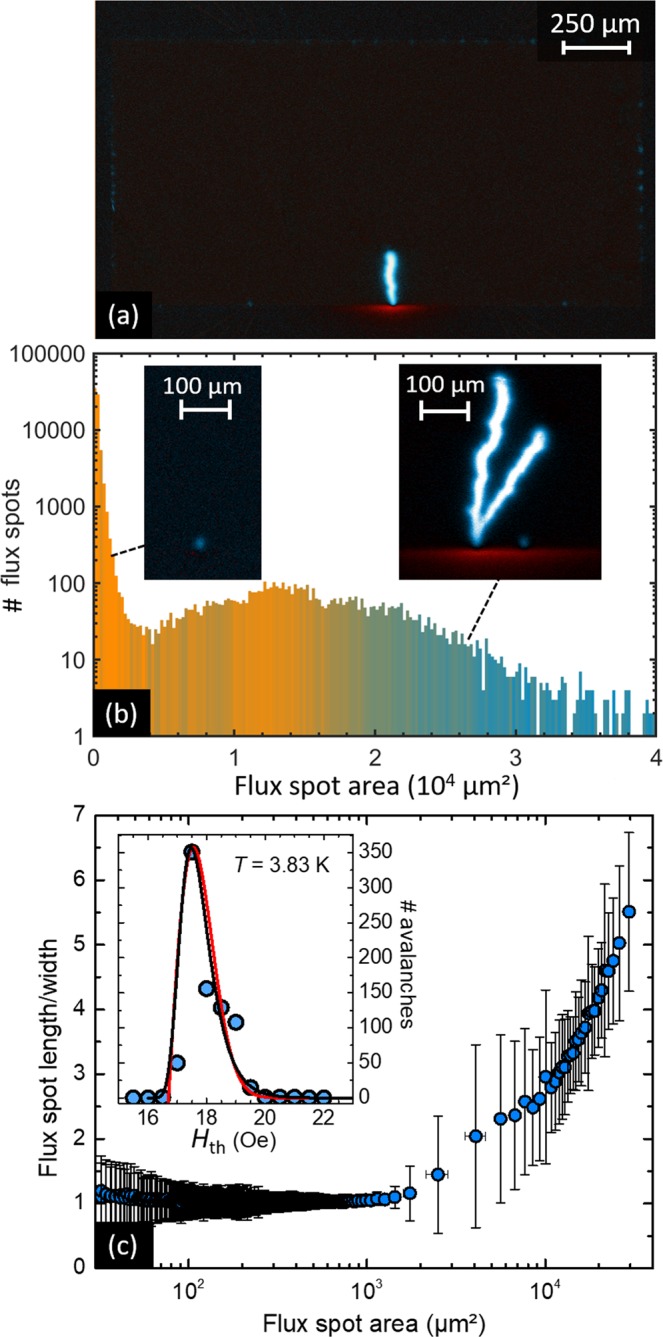
Statistical analysis of magnetic flux avalanches. (**a**) Differential MO image of the Nb film at *T* = 3.83 K. White-blue (red) regions correspond to positive (negative) field variations, while dark regions correspond to undetectable field variations. (**b**) Distribution of the avalanche sizes obtained from 79726 recorded events during 813 field sweeps from 14 Oe to 25 Oe. The graded color in the histogram indicates the mean field value at which the avalanches occur, from *H* = 17.75 Oe (orange) to *H* = 24.75 Oe (blue). The insets in panel (b) show that dynamically driven avalanches (left) exhibit a more rounded shape, whereas thermally driven avalanches (right) are elongated. In panel (c), the mean value of the aspect ratio *ε* of the avalanches is plotted as a function of the avalanche size. The dots correspond to an average over 100 avalanches and the error bars indicate the standard deviation. The inset shows the probability density function of the threshold field *H*_th_ at which thermally driven avalanches develop. The red and black lines correspond to fittings obtained with Eqs (1) and (2), respectively.

Magnetic flux changes are identified by subtracting consecutive images as illustrated in Fig. 2(a). The most salient feature of this differential image is the lightening-like magnetic flux burst at the lower center of the sample. The white-blue color indicates an increase of magnetic field intensity, whereas the red-white color points to a local decrease of the field magnitude resulting from decompression of magnetic field lines^26^. A careful inspection of this image shows small flux changes all along the border of the sample. The digital images allow for a precise estimation of the area covered by each magnetic flux change and thus the size of the avalanches. The frequency of appearance of a given avalanche size is presented in Fig. 2(b) in semi-log scale and with a color grade indicating the mean value of the magnetic field at which the avalanche has been triggered. Interestingly, a bimodal distribution is observed. Indeed, although the frequency of appearance of an avalanche tends to monotonously increase as the size of the event decreases (a general trend also reported for earthquake and granular avalanches distributions), a clear broad peak at large avalanche sizes is observed. This second peak is a unique fingerprint associated to avalanches initiated by a thermomagnetic instability which has no counterpart in the earthquake and granular avalanches analogies^14^. According to this description, the minimum in the size distribution indicates the transition between these two regimes and it could be used as a criterion to determine the first flux avalanche triggered by a thermomagnetic instability. Unfortunately, this criterion seems of little practical use since it requires as prerequisite to accomplish the whole statistical analysis.

A more functional approach to discriminate between dynamically and thermally driven avalanches is to look at the ratio *ε* = *L*/*W* characterizing the geometrical shape of the avalanche, where *L* and *W* are its length and width. Indeed, in the dynamically driven regime, the shape of the avalanche is dominated by thermal diffusion (${D}_{{\rm{m}}}\ll {D}_{{\rm{t}}}$) which is an isotropic process, thus giving rise to magnetic flux redistributions with $\varepsilon \gtrsim 1$. There is an inevitable asymmetry associated with the fact that avalanches develop in the direction of the magnetic flux gradient. In contrast to that, in the thermally driven regime where ${D}_{{\rm{m}}}\gg {D}_{{\rm{t}}}$, the shape of the avalanche is ruled by the magnetic diffusion which is highly directional (towards the center of the sample) and therefore elongated avalanches with $\varepsilon \gg 1$ are expected. These two extreme situations correspond well with the examples of avalanche shapes taken at either side of the minimum in the size distribution, as shown in the insets of Fig. 2(b). At the transition point between the two regimes, ${D}_{{\rm{m}}} \sim {D}_{{\rm{t}}}$, which roughly corresponds to *ε* = 2 at low temperatures. In Fig. 2(c), *ε* is plotted as a function of the avalanches size. The dots correspond to an average over 100 avalanches and the error bars indicate the standard deviation. The dynamically driven avalanche regime is clearly identified by the region where $\varepsilon  \sim 1$. By calculating the avalanche size distribution within this regime we find that it follows a power law with exponent −3.4, which falls within the range of values reported by previous Hall probe array measurements^27^ (see Supplementary Information). It is important to point out that previous studies by Treiber *et al*.^26,28^ have proposed alternative criteria to distinguish dynamic from thermomagnetic avalanches in highly disorder MgB_2_ thin films.

Based on the *ε* = 2 criterion separating the two regimes, we can now rigorously estimate the threshold magnetic field *H*_th_ at which the first thermally driven avalanche is triggered. The result of this analysis for *T* = 3.83 K is shown in the inset of Fig. 2(c). The alternative criterion corresponding to the local minimum of the size distribution provides very similar results. The obtained PDF shows a clear asymmetric shape. Previous investigation in MgB_2_ films repeating three runs under the same external conditions, suggested that dendrites tend to nucleate from fixed locations along the edge^29^. By identifying the loci of the avalanches triggered at each point of the distribution (Supplementary Information), we conclude that the multiplicity of these nucleation spots are the main cause of the broadening of the distribution.

To the best of our knowledge, there are no theoretical studies predicting the PDF of *H*_th_. Historically, the Weibull probability density function has been widely used to describe material or device failures^30^. This distribution is a weakest-link type distribution, meaning that the failure of the whole is dominated by the degradation rate of the weakest element (a nucleation point in our case). For instance, it properly describes dielectric breakdown, where the entire capacitor fails when a very localized region of the capacitor fails. The Weibull probability density function is defined by

1 $$P({H}_{{\rm{th}}})=\frac{\beta }{{H}_{0}}{(\frac{{H}_{{\rm{th}}}-{H}_{1}}{{H}_{0}})}^{\beta -1}{e}^{-{(\frac{{H}_{{\rm{th}}}-{H}_{1}}{{H}_{0}})}^{\beta }},$$

where *H*_0_ is referred to as the scale parameter, *H*_1_ is the location parameter, and *β* is referred to as the shape parameter.

Alternatively, the Gumbel distribution, given by

2 $$P({H}_{{\rm{th}}})=\frac{1}{{H}_{0}}\exp \left[\frac{-{(H}_{{\rm{th}}}-{H}_{1})}{{H}_{0}}-{e}^{\frac{{-(H}_{{\rm{th}}}-{H}_{1})}{{H}_{0}}}\right],$$

has been shown to be useful in predicting the probability that an extreme earthquake, flood or other natural disaster will occur^31^, as well as to describe the statistical distribution of phase slips in a long superconducting nanowire^32–34^. For the latter, the analogy is pertinent since the process of avalanche triggering starts from the contour of the sample, which can be regarded as a one dimensional nanowire.

An attempt to fit the experimentally determined probability density function with these two distributions is shown in the inset of Fig. 2(c). The red (black) curve corresponds to Eq. (1) [Eq. (2)] with the following parameters: *β* = 2, *H*_0_ = 1.2 Oe and *H*_1_ = 16.7 Oe (*H*_0_ = 0.5 Oe and *H*_1_ = 17.8 Oe). Although both Gumbel and Weibull distributions seem to properly account for the PDF of the first magnetic flux avalanche at low temperatures, we will show in the next section that a more complex probability distribution emerges at higher temperatures.

Note that the criterion based on *ε* should be temperature dependent. Indeed, as mentioned above, a typical dynamically driven avalanche does not exceed the flux front separating the critical state profile from the Meissner region. However, the distance between the sample border and the flux front increases with temperature. Therefore, it is expected that the value of *ε* separating dynamically from thermally driven avalanches also rises as temperature increases. It is important to point out that the observed dynamically and thermally driven avalanches may be influenced by the size of the sample, as reported in MgB_2_ films^35^. In addition, it is also expected that the threshold field for triggering flux avalanches decreases with increasing the field ramp rate^36^. However, previous studies by Nowak *et al*.^14^ showed that the avalanche activity in Nb rings remains unaffected for rates ranging over four decades from 0.002 Oe/s to 20 Oe/s. In the present experiments, a maximum field ramp rate of about 200 Oe/s has been used.

## Threshold magnetic field of thermomagnetic breakdown

**Figure 3:**
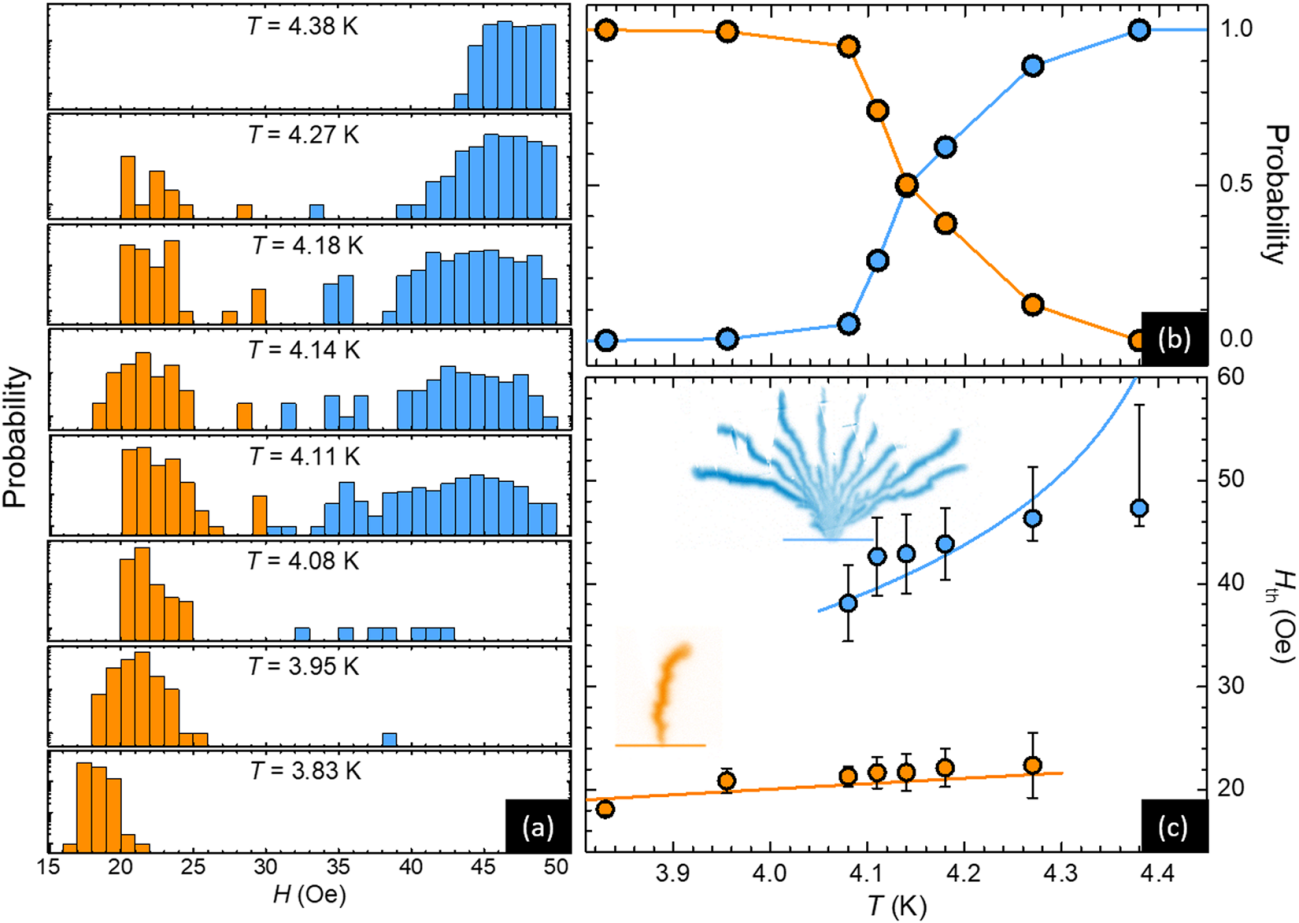
Threshold field for triggering thermally driven avalanches. (**a**) Probability density function of the threshold field *H*_th_ in semi-log scale for several temperatures *T*. The bimodal distribution exhibits a peak at low fields associated to the development of finger-like avalanches (in orange), and a broader peak at higher fields corresponding to dendritic avalanches (in blue). (**b**) Probability that the first avalanche is of filamentary type (orange dots) or branching type (blue dots) as a function of *T*. (**c**) *H* − *T* diagram *H*_th_(*T*) showing the measured and computed transition lines for the two different avalanche morphologies.

It has been reported that thermally driven avalanches can exhibit either a finger-like morphology at low temperatures or a branching structure at higher temperatures^37^. The question arises as to how this transition takes place and how sharp this crossover is. Figure 3(a) summarizes the resulting PDF of *H*_th_ for several temperatures in semi-log scale. The lowest temperature of 3.83 K is limited by the base temperature of our cryostat, whereas above 4.40 K, no avalanches were detected. The color code of the histogram indicates the morphology of the first thermally driven avalanche, with orange for the finger-like type and blue for the dendritic branching. At *T* = 3.83 K, 100% of the first avalanches detected are finger-like, whereas at *T* = 4.38 K, 100% of the first avalanches detected are of branching type. In between these two extrema, the PDF shows two well distinguished distributions. The amplitude of the peak corresponding to filamentary avalanches progressively decreases whereas the peak associated to dendritic flux penetration becomes more dominant as temperature increases. This is more clearly seen in Fig. 3(b) where the probability that the first avalanche is of one type or another is plotted as a function of temperature. A transition between these two regimes is observed close to *T* = 4.15 K. It has been argued, based on numerical simulations^38^, that this transition may be associated to a change in the lateral heat diffusion. The sharpness of the observed transition is at odds with this interpretation since no abrupt change in the lateral heat diffusion is expected. The authors of ref.^38^ also showed that the morphology significantly depends on the initial background flux penetration depth prior to the triggering of the avalanche. This seems to be in agreement with our observation that dendritic avalanches take place at higher fields and correspondingly with a larger flux penetration depth than finger-like avalanches.

A *H* − *T* diagram depicting the transition lines corresponding to the threshold magnetic field *H*_th_ at which the first thermally driven avalanches are triggered is shown in Fig. 3(c). The lower (orange) dots indicate the transition to finger-like avalanches and the upper (blue) dots the transition to dendritic avalanches. The observed small variation of the threshold field at low temperatures is a general characteristic also reported in MgB_2_ films^39^ and tapes^40^. Closed-form expressions for the threshold magnetic field were recently derived for different regimes where the onset of avalanches is delayed either by heat diffusion through the superconducting sample, by the specific heat of superconductor or by heat evacuation through the substrate^10^.

At low temperatures, the heat evacuation coefficients are sufficiently low and the system can be considered in an adiabatic regime where the instability is solely prevented by the specific heat of the superconductor. In this case the threshold field is given by^10^

3 $${H}_{{\rm{t}}{\rm{h}},C}=\sqrt{\frac{2\,{C}_{{\rm{N}}{\rm{b}}}({T}_{c}-T)t}{\pi \,{\mu }_{0}\,w}},$$

where *t* is the Nb film thickness, *w* the half width of the film and *C*_Nb_(*T*) = *C*_Nb,0_(*T*/*T*_c_)^3^ the specific heat of the superconductor. Fitting the low field data points (in orange) with Eq. (3), we find *C*_Nb,0_ = 5.5 × 10^5^ J.K^−1^.m^−3^, which is comparable to typical values found in the literature^41^, and observe a reasonable agreement between theoretical expression and experimental data.

At higher temperatures, the heat dissipation through the substrate becomes the delaying parameter and the transition line is given by

4 $${H}_{{\rm{t}}{\rm{h}},h}=\frac{t\,{j}_{{\rm{c}}}}{\pi }\,{\rm{a}}{\rm{r}}{\rm{c}}{\rm{t}}{\rm{a}}{\rm{n}}{\rm{h}}(\frac{h({T}_{{\rm{c}}}-T)}{n\,w\,t\,{j}_{c}\,{\mu }_{0}\,{\dot{H}}_{a}}),$$

where *Ḣ*_a_ is the time derivative of the applied magnetic field during a step of the sweep, *j*_c_ = *j*_c,0_(1−*T*/*T*_c_) is the critical current density, *h* = *h*_0_(*T*/*T*_c_)^3^ is the coefficient of heat transfer between the superconductor and the substrate and *n* is the critical exponent. Using *Ḣ*_a_ = 200 Oe.s^−1^ and^41^ *j*_c,0_ = 1.5 × 10^11^ A.m^2^, we find *h*_0_/*n* = 2.8 × 10^−1^ W.K^−1^.m^2^ for the blue curve in Fig. 3(c). Note that the last two experimental points underestimates the *H*_th_ value due to the set-up limitation to apply magnetic fields higher than 50 Oe. The clear mismatch between the experimental data and the theoretical expression can be explained by the large uncertainty in the parameters at play and by the fact that Eq. (4) assumes that the local temperature remains close to the bath temperature, which has been shown to be a strong hyphothesis^42^. In that sense, the solid line corresponding to *H*_th,*h*_ should be taken with caution and interpreted as a general trend expected from the model. Both Eqs (3) and (4), take into account the demagnetization factor. From the measured *H*_th_, it is possible to roughly estimate the local field as ${H}_{{\rm{local}}} \sim {(w/t)}^{\mathrm{1/2}}{H}_{{\rm{th}}}$, where *w* is the half width of the sample and *t* its thickness. Since the geometrical prefactor is a large figure (~70), there is a substantial difference between *H*_local_ and *H*_th_. However, nearly the same geometrical factor applies for every datapoint, and therefore the trend observed in Fig. 3 remains the same irrespective of plotting the data against *H*_local_ or *H*_th_.

We should emphasize that the two transition lines *H*_th_ seem to end at a precise point, a feature that has not been anticipated by the theoretical investigations. Nevertheless, their reasonable agreement with the measured *H*_th,*C*_ and *H*_th,*h*_ suggests that the observed abrupt transition between the two morphology regimes arises from a change in the mechanism of heat dissipation. Further systematic experimental investigation in other superconducting materials and substrates will certainly help to corroborate this finding.

## Conclusion

To summarize, we provide a clear quantitative criterion based on magneto-optical imaging for distinguishing dynamically driven flux avalanches from those originating from thermomagnetic instabilities without the need of thermography or time resolved measurements. This technique permits to surpass previous studies based on Hall probe arrays and global magnetization measurements which conceal information about different nucleation spots in the sample, size and shape of individual avalanches. Based on the proposed quantitative criterion, we are able to determine the probability density function of the threshold field of thermomagnetic breakdown, track its temperature dependence with unprecedented resolution and unveil the details of the transition from filamentary to dendritic branching avalanches. The reasonable agreement of the two measured *H*_th_(*T*) transition lines with the recently developed theoretical model suggests that a change of damping mechanism from in-plane heat diffusion to heat transfer to the substrate is at the origin of the observed two regimes. The studied system is non-destructive, which makes it to stand out from previous investigations of the triggering statistics of avalanche type events. This, in turn, allows to unequivocally assess the probability density function without being affected by the inevitable dispersion associated with an ensemble of replicas.

## Supplementary information

Statistics of thermomagnetic breakdown in Nb superconducting films: Supplementary Information

## Data Availability

All data used in this article is presented in the manuscript and in the Supplementary Materials.
