# Publisher Correction: Statistics of thermomagnetic breakdown in Nb superconducting films

**DOI:** 10.1038/s41598-020-60498-1

**Published:** 2020-02-27

**Authors:** S. Blanco Alvarez, J. Brisbois, S. Melinte, R. B. G. Kramer, A. V. Silhanek

**Affiliations:** 1 Experimental Physics of Nanostructured Materials, Q-MAT, CESAM, Université de Liège, B-4000 Sart Tilman, Belgium; 2 Institute of Information and Communication Technologies, Electronics and Applied Mathematics (ICTM), Institut de la Matière Condensée et des Nanosciences (IMCN), Université catholique de Louvain, 1348 Louvain-la-Neuve, Belgium; 3 Institut Néel, CNRS, Université Grenoble Alpes, 38042 Grenoble, France

Correction to: *Scientific Reports* 10.1038/s41598-019-39337-5, published online 06 March 2019

The Supplementary Information file originally published with this Article was incorrectly published as a ‘.tex’ file. The correct Supplementary Information file now accompanies the Article.

