# Supplementary material for: Statistics of thermomagnetic breakdown in Nb superconducting films
Source: Sci Rep. 2019 Mar 6;9:3659. doi: 10.1038/s41598-019-39337-5 (PMC6403392; doi:10.1038/s41598-019-39337-5)
Supplement: Supplementary file 1 — Statistics of thermomagnetic breakdown in Nb superconducting films: Supplementary Information [file 41598_2019_39337_MOESM1_ESM.pdf]

Statistics of thermomagnetic breakdown in Nb superconducting films: Supplementary Information

S. Blanco Alvarez,[1] J. Brisbois,[1] S. Melinte,[2] R. B. G. Kramer,[3] and A. V. Silhanek[1,*]

[1] *Experimental Physics of Nanostructured Materials, Q-MAT, CESAM, Université de Liège, B-4000 Sart Tilman, Belgium.*

[2] *Institute of Information and Communication Technologies, Electronics and Applied Mathematics (ICTM), Institut de la Matière Condensée et des Nanosciences (IMCN), Université catholique de Louvain, Louvain-la-Neuve, Belgium.*

[3] *Institut Néel, CNRS, Université Grenoble Alpes, Grenoble, France.*

(Dated: November 9, 2018)

I. INFLUENCE OF THE NANOHEATERS ON THE MAGNETIC FLUX PENETRATION

In the adopted sample geometry, the nanometric thermal elements are located in the vicinity of the rectangular Nb film. The four nanoheaters are made of the same superconducting material as the rectangular Nb film and therefore, it is pertinent to wonder about their influence on the local penetration of flux in the Nb film. Indeed, if no current circulates in the nanoheater, the screening Meissner currents will expel the magnetic field from the device, giving rise to a concentration of magnetic flux lines in the 1 μm gap separating the nanoheater from the border of the Nb film. It is precisely the presence of this screening effect which permits to visualize the Nb wires of the inactive nanoheaters in the magneto-optical image shown in Fig. 1.

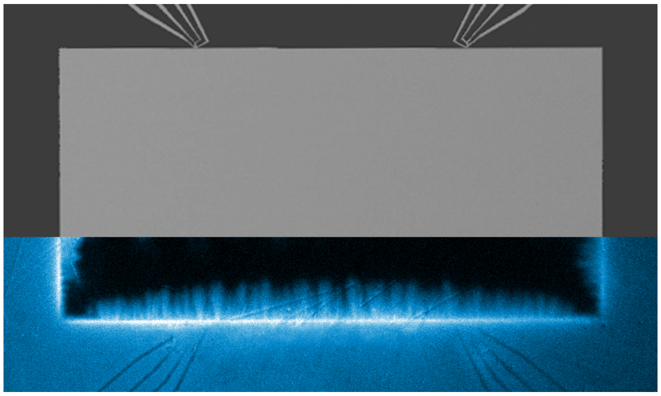

FIG. 1. Magneto-optical image of the sample's magnetic flux penetration at 7 K for a magnetic field H applied perpendicularly to the sample, with $H = 18.5$ Oe.

Note first that if the central part of the nanoheater has been modified by electromigration, it becomes a normal metal and therefore interrupts the Meissner currents and their associated effects. If no electromigration process has been practiced on the heating element, the flux focusing effect[1] has to be taken into account. Simple arguments based on the field distribution around a superconducting film[2] indicate that such perturbations of the magnetic field can be ignored. Indeed, on the one hand the magnetic field at the border of a Nb strip $B_z \sim (w/d)^{1/2} B_a$ of width w, largely exceeds the applied field B_a. On the other hand, the field perturbation with respect to B_a decreases rapidly within a distance $\sim w/4$ from the border of the sample. Based on the fact that the nanoheater width is about 1 μm and the distance from its border to the border of the film is on the same order, the nanoheater produces a negligible perturbation of the magnetic field at the border of the film.

We have verified experimentally that no influence of the heating elements is observed by inspecting the MOI obtained at 7 K, for perpendicular applied fields from 0 Oe to 50 Oe by steps of 0.25 Oe. At this temperature, no thermomagnetic instabilities take place and the flux penetration tends to follow the Bean profile, as shown in the magneto-optical image of Fig. 1 recorded at $H = 18.5$ Oe.

It is worth emphasizing that there are no effects associated to the field generated by the large currents applied to the heater since when the heating elements are active, no external field is applied and no measurements are acquired.

II. CRITICAL EXPONENT IN THE DYNAMICALLY DRIVEN FLUX AVALANCHE REGIME

When a magnetic field is applied to the superconducting film, the system can relax the magnetic pressure built up at the sample border either through dynamically driven avalanches (i.e. non thermal) or via thermomagnetic avalanches. As shown in Fig. 2(b) of the main text, the dynamically driven avalanche regime typically correspond to small size events in the bimodal distribution. It has been long argued that within this regime signatures of self-organized criticality could be observed.

According to the theory of self-organized criticality, the probability P of an avalanche involving N vortices follows a power law $P(N) = N^{-\alpha}$, reflecting the scale invariant nature of the phenomenon. A critical exponent $\alpha = 3$ for Nb foils has been obtained by Altshuler and co-workers from combined MOI and Hall magnetometry[3]. In Fig. 2, we plot the distribution of the number of avalanches as a function of their size in the range corresponding to

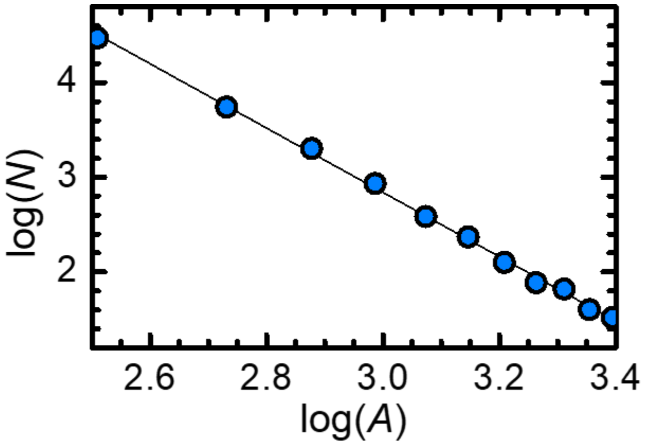

FIG. 2. Distribution of the number N of avalanches as a function of their size A in μm^2. The black line corresponds to a linear adjustement and gives a critical exponent $\alpha = 3.4$.

the first mode of the distribution in Fig. 2(b) of the main text. A linear fitting, represented by the black line in the log-log graph, gives a critical exponent $\alpha = 3.4$, which is close to the value reported in previous studies[3].

III. SPATIAL DISTRIBUTION OF THERMOMAGNETIC FLUX AVALANCHES

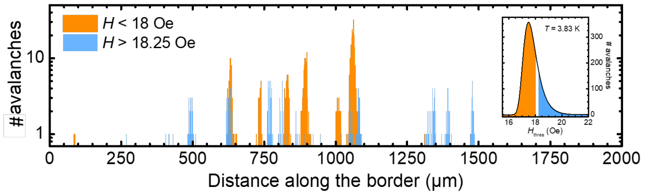

FIG. 3. Spatial distribution of the first flux avalanches triggered along the bottom edge of the sample at $T = 3.83$ K. The data in orange corresponds to avalanches appearing at a field $H < 18$ Oe, while the blue bars correspond to those triggered at $H > 18.25$ Oe.

The finite width of the distribution of the threshold field H_{th} at which avalanches are triggered (inset of Fig. 2(c)) originates from the distribution of nucleation points along the sample border. This is clearly illustrated in Fig. 3 showing the number of avalanches detected along that border in 1 px= 1.468 μm wide intervals. Notice that the activity is higher at the center of the sample and diminishes towards the corners as a consequence of the fact that avalanches are only triggered if the Bean profile exceeds a characteristic length[4].

Interestingly, by plotting in orange the avalanches triggered at $H_{th} < 18$ Oe and in blue those appearing for $H_{th} > 18.25$ Oe, we can see that nucleation points with low threshold fields do not overlap much with those having high threshold fields. It is tempting to associate these spatial distribution of avalanches to a spatial distribution of border defects. As we will demonstrate in the next section, this interpretation might be misleading since thermomagnetic avalanches tend to avoid border defects.

IV. ANTICORRELATION BETWEEN DYNAMICALLY AND THERMALLY DRIVEN AVALANCHES

It is widely asummed that thermomagnetic flux avalanches should preferably nucleate at the location of defects, due to the large electric fields and the vortex traffic found at those points[5–7]. However, it has been recently experimentally shown that defects might actually have the opposite effect, i.e. thermomagnetic avalanches will be less likely to occur in their vicinity[8]. A possible argument is that defects act as flux faucets and favour dynamically-driven flux penetration, thus releasing the magnetic flux pressure accumulated at the sample border.

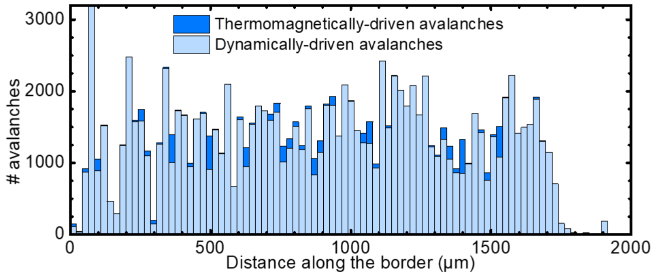

FIG. 4. Spatial distribution of dynamically-driven (in light blue) and thermomagnetically-driven (in dark blue) flux avalanches along the bottom edge of the sample at $T = 3.83$ K.

In order to dig further on this issue, we have analyzed the spatial correlation between dynamically-driven and thermomagnetically-driven avalanches. Fig. 4 shows the spatial distributions of dynamically-driven (light blue) and thermomagnetically-driven (dark blue) flux avalanches triggered at the bottom edge of the Nb superconducting film at $T = 3.83$ K. Note that unlike in Fig. 3, here all thermomagnetically driven avalanches are included. A close inspection to Fig. 4 indicates that thermomagnetic avalanches are mainly active where dynamically-driven avalanches exhibit a minimum activity. This finding seems to corroborate previous reports[8] showing that dynamically-driven and thermomagnetically-driven avalanches are spatially anticorrelated.

* asilhanek@uliege.be

[1] A. A. B. Brojeny, Y. Mawatari, M. Benkraouda, and J. R. Clem, Supercond. Sci. Technol **15**, 1454 (2002).

[2] J. R. Clem and A. Sanchez, Phys. Rev. B **50**, 9355 (1994).

[3] E. Altshuler, T. H. Johansen, Y. Paltiel, P. Jin, K. E. Bassler, O. Ramos, Q. Y. Chen, G. F. Reiter, E. Zeldov, and C. W. Chu, Phys. Rev. B **70**, 140505 (2004), cond-mat/0208266.

[4] D. V. Denisov, D. V. Shantsev, Y. M. Galperin, E.-M. Choi, H.-S. Lee, S.-I. Lee, A. V. Bobyl, P. E. Goa, A. A. F. Olsen, and T. H. Johansen, Phys. Rev. Lett. **97**, 077002 (2006).

[5] T. Schuster, H. Kuhn, and E. H. Brandt, Phys. Rev. B **54**, 3514 (1996).

[6] T. Schuster, M. V. Indenbom, M. R. Koblischka, H. Kuhn, and H. Kronmüller, Phys. Rev. B **49**, 3443 (1994).

[7] J. I. Vestgården, D. V. Shantsev, Y. M. Galperin, and T. H. Johansen, Phys. Rev. B **76**, 174509 (2007).

[8] J. Brisbois, O.-A. Adami, J. I. Avila, M. Motta, W. A. Ortiz, N. D. Nguyen, P. Vanderbemden, B. Vanderheyden, R. B. G. Kramer, and A. V. Silhanek, Phys. Rev. B **93**, 054521 (2016).
